# Managing Exposure to Benzene and Total Petroleum Hydrocarbons at Two Oil Refineries 1977–2014

**DOI:** 10.3390/ijerph15020197

**Published:** 2018-01-24

**Authors:** Tapani Tuomi, Henna Veijalainen, Tiina Santonen

**Affiliations:** Finnish Institute of Occupational Health (FIOH), Topeliuksenkatu 41 B, P.O. Box 40, Työterveyslaitos, FI-00032 Helsinki, Finland; henna.veijalainen@ttl.fi (H.V.); tiina.santonen@ttl.fi (T.S.)

**Keywords:** oil refining, hydrocarbon exposure, best practices

## Abstract

Air concentrations of and inhalation exposure to total petroleum hydrocarbons (TPH) and benzene was monitored separately at two oil refineries from 1977 to 2014. Prevention policies and control measures that may explain changes were surveyed. The aim was to evaluate how the application of of Occupational Health and Safety Assessment Series OHSAS 18001.04 principles as well as Environmental protection Agency EPA and European Oil Company Organisation for Environment, Health and Safety CONCAWE practices have influenced air concentrations. Benzene air concentrations declined in 11 of 17 units, six of which were associated with declining exposures. Benzene air concentrations declined across all units on average by 46%. This amounts to an average yearly decline of 1.7%. TPH air concentrations declined in 10 of 17 units, seven of which were associated with declining exposures. The average decline in TPH air concentrations was 49%, corresponding to 1.3% per year. As a result, average working day exposure in 10 of 17 units have declined significantly and today, benzene and TPH exposure in most units are well below 10% of the current Occupational Exposure Limit (OEL_8h_:s). A decline in air concentrations have coincided with consistent implementation of control measures. Such measures include on-line monitoring of leaks; benzene recovery; floating container roofs; improved valves and seals; hermetic pumps; recovery of loading gases and instalment of torches in terminals; cutback in coke combustion; a new production line spanning directly from the dock to aromatics production; and recovery of loading gases in the doc. Other tools in exposure management include personal leak monitors, on-line measurements, monitoring campaigns, risk assessment, and availability and user training of protective equipment. However, improvements are still needed. Hydrocarbon or benzene air concentrations have not declined in 8 of 17 units, in some of which concentrations exceed 10% of the relevant OEL_8h_ value. In addition, for benzene even 10% of the current OEL, 0.1 ppm, might still possess a risk. With this in mind, methods to estimate exposure at the refineries need to be improved to enable measuring benzene concentrations <0.1 ppm. Shut downs of the refinery have been associated with peaks in exposure concentrations. Consequently, effort should be placed on safe working methods pertaining to shutdowns. Also, the connection and detachment of hoses continues to be problematic from the point of view of controlling exposure.

## 1. Introduction

Mineral oil refineries handle vast amounts of raw oil and distillates as well as other oil-based products, mostly outdoors. During continuous operation, many of the unit operations as well as the transfer of streams between units is associated with stray leaks or sampling and maintenance operations, leading to exposure of personnel to petrochemicals [1,2]. In addition, during turnarounds and periodic shutdowns, maintenance operations may lead to high exposures to hydrocarbons [3,4]. Main exposure agents include, for example, benzene, toluene, ethylbenzene, xylenes, methyl *tert*-butyl ether (MTBE), *tert*-amyl methyl ether (TAME), in addition to other aliphatic or aromatic hydrocarbons with a carbon number between C5 and C35 [5]. As a whole, these are often referred to as total volatile hydrocarbons or total petroleum hydrocarbons (TPH).

Benzene has been classified by the International Agency for Research on Cancer (IARC) as carcinogenic to humans (Group 1 classification), based on the etiology of leukemia. In addition, benzo(a)pyrene is categorized to Group 1, based on lung tumourigenesis and other genotoxic effects. Some other TPH compounds or petroleum products, such as gasoline, are considered to be probably or possibly carcinogenic to humans (IARC Groups 2A and 2B, respectively). Most of the other TPH compounds and products are considered not classifiable (Group 3) by IARC. In addition to cancers, inhalation exposure to TPH compounds may be associated with symptoms of the central nervous system, in addition to headaches, dizziness and irritation of eyes and the respiratory tract [5]. 

Based mainly on its genotoxic properties and with the gathering of novel dose-response data [6,7], the occupational limit value of benzene in Finland has been decreased from 10 ppm to 1 ppm during the time-span of the present study, 1977–2014 (Table 1). Unless the aromatics content exceeds 25% and/or hexane content exceeds 1%, exposure to TPH compounds in Finnish refineries—measured as total volatile hydrocarbons—are presently generally compared to the Occupational Exposure Limit (OEL_8h_) of “Category two” solvent petrols, 200 mg/m^3^. Based on, for instance, neurotoxic, irritatory, liver toxic, reproductive and developmental effects [5], the OEL_8h_ of these solvent petrols in Finland has been lowered stepwise from 1150 mg/m^3^ to 200 mg/m^3^ between 1977 and 2014 (Table 1).

During regular operation conditions, exposure in modern refineries to benzene and other hydrocarbons is mostly below Finnish OEL_8h_ values or corresponding values stipulated by, for instance, EU, Occupational Safety and Health Administration (OSHA), National Institut Occupational and Security Health (NIOSH) or American Conference of Governmental Industrial Hygienists (ACGIH) [1,8]. Exposures of in-house personnel to benzene has been reported to be lower during turnarounds and shutdowns than routine production [4,9,10]. During normal operations, high benzene or TPH exposures have been recorded in the loading and unloading of raw oil and products, as well as sample collection, installing and removing of blinds, tank cleaning, pipefitting and welding, waste treatment, and the cleaning of equipment [4,8]. During shut downs and turnarounds, plumbing, scaffold work, welding, drain fitting, and general field work can be challenging with respect to managing benzene and TPH exposure [3,8].

### 1.1. Exposure to Benzene and Relevant Health Implications

Benzene concentration of crude oil can be up to 4 g/L [11]. Within the EU, the maximum benzene concentration in petrol is currently 1% (EU DIR 98/70/EY). Benzene is formed during heating and incomplete combustion of organic materials and as such, occurs ubiquitously in non-rural environments in concentrations below ca. 1.5 ppb [12].

Absorption of benzene through the lungs is rapid. The proportions absorbed are inversely related to the exposure concentration [6,7]. At exposure levels of ~1–10 ppm approximately 50% of benzene is absorbed [13]. Short term (60 min) exposure to 50–150 ppm may cause headache, lassitude and weariness [14]. From epidemiological studies there is clear evidence on the association between benzene exposure and acute myeloid leukemia, acute non-lymphocytic leukemia, as well as myelodysplastic syndrome [15]. It has also shown to cause non-malignant haematotoxic effects, including leukopenia [16].

### 1.2. Background and Aims of the Study

The present study was initiated by the finding that workers employed at the refineries during 1967–1982, had a 2–3 fold risk of attaining kidney cancer, when compared to the general (reference) population [17,18], and that this increase may be associated with exposure to hydrocarbons (30–200 °C) and/or benzene [18]. These previous results reflect exposures up to the 1980s. As current exposures to hydrocarbons, including benzene, may be different, we wanted to examine air concentrations as well as inhalation exposure to benzene and TPH during recent decades (1977–2014). Particular interest was directed to the time-dependent development of concentrations and to control measures that may explain changes, when found. As all possible improvements are based on the safety management system of the company, we attained as much information as possible about measures taken that were initiated by corporate occupational health and safety strategies.

In short, the aim was to evaluate how the application of Occupational Health and Safety Assessment Series (OHSAS) 18001 and 18004 principles as well as Environmental protection Agency (EPA) and European Oil Company Organisation for Environment, Health and Safety (CONCAWE) exposure management practices have influenced air concentrations of and inhalation exposure to the examined agents.

## 2. Materials and Methods

### 2.1. The Company

The Neste Ltd. (Espoo, Finland) refineries in Porvoo and Naantali produce fuels, solvents, bitumen and lubricants, including the most important oil products. The refinery in Naantali was put into use in 1957, while the Porvoo refinery has operated since 1966. The Naantali refinery was originally designed to refine fuels, lubricants and bitumen products from crude oil, with an annual capacity of 800,000 tons. In 2014, the total capacity of oil products and renewable products of the two refineries were 15 million and 2 million tons per year, respectively. In 2014, the main product groups were motor gasoline and gasoline components (26%), diesel fuel (37%), renewable fuels (12%) and heavy fuel oil (8%).

The company has a certified Occupational Health and Safety management system based on OHSAS 18001 (2007) [19]. In addition to risk assessment and management, key components of safety management at the refineries include a work permit practice, safety discussions, safety observation tours, and inspections. Workers, including subcontractors, are yielded work permits only when they have been sufficiently trained in safe working methods. At the onset of a new production unit, relevant exposures are measured to either confirm the sufficiency of exposure management measures or assist in the planning of further measures.

### 2.2. Basic Exposure Monitoring Programmes, Online Monitoring of Emissions

Exposure monitoring in the refineries is based on annual monitoring programmes planned by HSE Services in cooperation with the manufacturing units and the occupational health care unit. HSE Services gathers all occupational hygiene samples in the refineries. The timetable of exposure monitoring following initial measurements at a new unit, are dependent on the concentrations in the initial measurements as well as the implementation schedule of exposure management measures. In each case, measurements are followed up within five years.

In addition to the units and working tasks included in the basic monitoring programs, exposure monitoring is performed in conjunction with accidents or production disturbances or changes with a potential to increase exposure.

To prevent excessive exposure and detect leaks, hydrocarbons and various flammable gases, carbon monoxide, hydrogen sulfide and oxygen are continually measured on-line using personal Leak Detection And Repair monitors (LDAR monitors) carried by all workers in the refinery area [20,21]. In addition, ca. 300 monitors are installed in stationary points in the refinery area, critical with respect to exposure and/or leaks. 

Benzene concentrations in the production area are currently monitored by an on-line gas chromatograph connected to 11 stationary sampling points. The measuring range of the chromatograph is 0.5 ppm. The data is visible to the unit operators. The alarm limit pertaining to these measurements is 0.3 ppm. 

### 2.3. Sample Collection and Analysis in the Present Study

Altogether 4588 samples were taken and analysed by Neste Ltd. HSE Services during the relevant time span (1977–2014) (Table 2 and Table 3). Out of these 4588 samples, 1534 (33%) describe average working day exposure as measured from the breathing zone of workers not wearing respiratory protective equipment (Table 3). The samples were collected indoors or outdoors, either from stationary points or from the breathing zone of workers at 18 different working units. The measurements included tasks from all units with the aim to measure air concentrations or to distinguish average 8 h (or 12 h) inhalation exposure during normal work shifts at the refineries. The samples were taken mainly in association with annual monitoring programs. Results describing average working day exposure were treated separately (Table 3). All samples were withdrawn by occupational hygienists or technicians from Neste HSE Services.

The results that include breathing zone samples outside of respirators as well as stationary point samples not necessarily descriptive of average 8 or 12 h working day exposure are referred to as “air concentrations” throughout. Samples collected from stationary points were collected at a height of 1.5 m above the floor or ground.

Samples were withdrawn to SKC-226-01 charcoal adsorbent tubes at a sampling rate of ca. 0.1 L/min using SKC 222 (SKC Inc., Eighty Four, PA, USA) or Gilian LFS-113 DC (Sensidyne LP, St. Petersburg, FL, USA) sample pumps, or other SKC or Gillian (Sensidyne LP) pumps with a similar sampling range (0.05–0.2 L/min). Aliphatic and aromatic hydrocarbons with a boiling point of ca. 35–220 °C, were extracted from the adsorbent in 4 mL glass tubes with 1.5 mL of carbon disulfide. Main components in the samples were dependent on the production unit and work-task examined and included for example benzene, toluene, ethylbenzene, xylenes, methyl *tert*-butyl ether (MTBE), *tert*-amyl methyl ether (TAME), in addition to aliphatic hydrocarbons. Samples were analysed using a gas chromatograh equipped with Flame Ionization Detectors (FID) and one or two columns. If two columns were used, they were of dissimilar polarity characteristics: for instance, HP-InnoWax (30 m × 0.32 mm i.d., 0.5 µm; Agilent Technologies, Santa Clara, CA, USA) and HP 5 (30 m × 0.32 mm i.d., 1 µm; Agilent Technologies).

Compounds were identified and quantitated using pure calibrators at a minimum of three different concentrations, spanning the concentration range of 0.01–10 µL/sample. The Limit Of Quantitation (LOQ) of the analysis corresponded to the smallest calibrator and was generally ca. 8 µg/sample. For benzene, the detection limit was 13 µg/sample, corresponding to ca. 0.1 ppm for a sample withdrawn during a workday (7 h) with a sampling rate of 100 mL/min. Blanks and control standards (one in each run) were spiked onto sorbent tubes and prepared analogically to the samples.

Samples were kept refrigerated (−20 °C) until analysis. The samples were analysed with an HP or Agilent 6890 gas chromatograph operated using MS Chemstation software (Hewlett-Packard, Palo Alto, CA, USA), and equipped with two FID detectors, one for each column. The operating conditions were set and the results calculated as described in the reference methods NIOSH 1500–1501. 

### 2.4. Testing the Time-Dependency of the Concentration Measurement and Calculating the Change during the Measured Time Interval

A linear regression analysis was performed on the sampling date (x) and concentration (y) data. The Pearson correlation coefficient was tested against its sample-size dependent *p*-value (critical value, 95% level of significance, two tailed test) as described by Warner (2013) [22]: ρ_x,y_ = √(*t*^2^/(*t*^2^ + *n* − 2)) at the *t*-test critical value (*t*) corresponding to the relevant degree of freedom. The test was performed to distinguish units where the concentration of benzene and/or total volatile hydrocarbons significantly increased or decreased as a function of the date of sampling during the time-interval of the measurements, from those where a statistically significant change could not be verified.

If a linear relationship (sampling date vs. concentration) was established, the proportional (%) change in concentrations was estimated by comparing the value at the starting point of the regression interval (x_1_,y_1_) to the concentration at the end point (x_2_,y_2_):%Ch = (y_1_ − y_2_)/y_1_ × 100.

### 2.5. Production Lines 1–5

The production lines (1–5) differ in their output and also in the raw materials used and, consequently, in their benzene emissions as well as the amount and profile of volatile hydrocarbons emitted to their immediate environment. Production lines 1–3 produce mainly gasoline and production line 4 mainly diesel fuel. Production line 5 yields bitumen, small engine gasoline and solvents, in addition to diesel fuel. Production lines 1–4 are situated in Porvoo, while line 5 is in Naantali.

## 3. Results 

### 3.1. Production Lines 1–5

**Figure 1:**
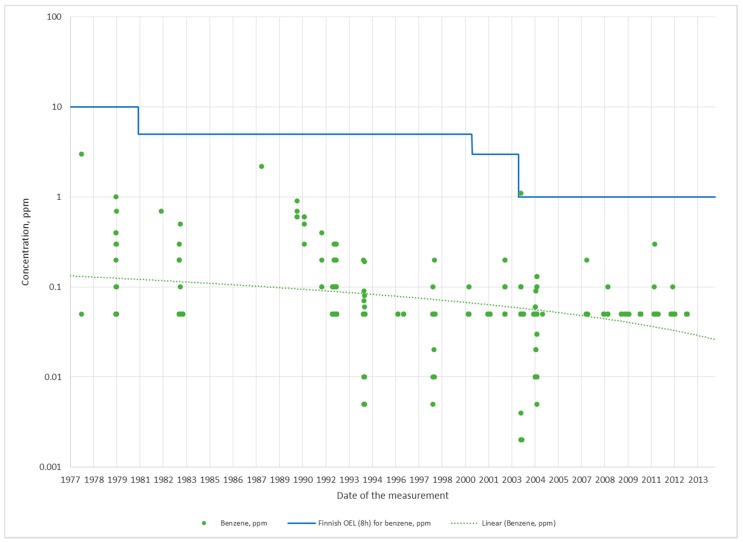
Benzene measurements from production lines 1–4 in Porvoo included in annual monitoring campaigns (average exposure during normal working shifts).

**Figure 2:**
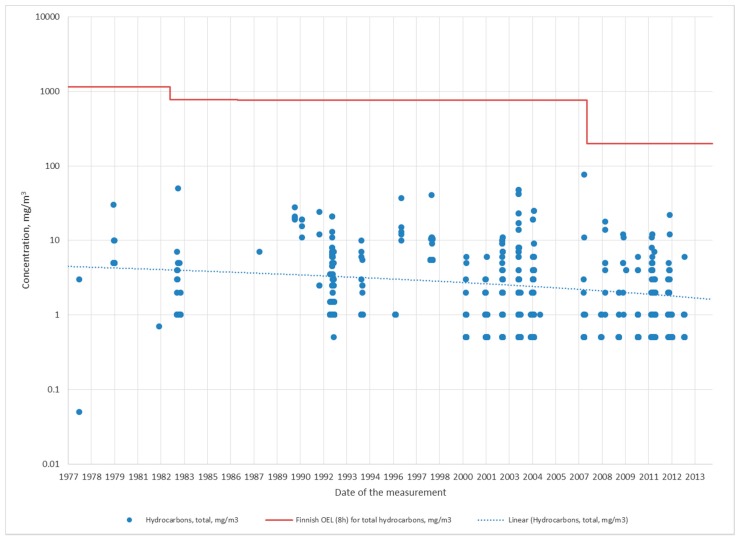
Hydrocarbon measurements from production lines 1–4 in Porvoo included in annual monitoring campaigns (average exposure during normal working shifts.

Characteristic for the production lines in Porvoo (lines 1–4) is that while accidents, shut downs and operating disturbances have on occasion led to high benzene and TPH concentrations, the air concentrations during normal working shifts have been fairly low during all of the years studied (Figure 1 and Figure 2 and Table 2), with the inhalation exposures for the most part being close to or similar to ambient concentrations (Table 3). Average and median benzene air concentrations during 37 years in production lines 1–4 were 0.36 ppm and 0.05 ppm, respectively, while the average and median TPH concentrations were 17 mg/m^3^ and 1 mg/m^3^, respectively. The medians of the air concentrations amount to 5% (benzene) and 0.5% (TPH) of the current OEL_8h_ values, 1 ppm for benzene and 200 mg/m^3^ for TPH.

In the Naantali production line (line 5), the average and median benzene air concentrations have been of the same magnitude as in the Porvoo production lines, namely 0.35 and 0.05 ppm, respectively. The average and median TPH concentrations have, however, been notably higher in production line 5 than in other production lines: average 56 mg/m^3^, median 15 mg/m^3^ (7.5% of the current TPH OEL_8h_ value). In production lines 1–5, benzene concentrations have very rarely reached concentrations that necessitate the use of respiratory protective equipment. In fact, in production lines 2 and 4, no such incidents have been monitored (Table 2). 

In production lines 1, 3 and 5, the decline in measured benzene air concentrations have been steep starting with 1977. In line 2, we see a clear but nonetheless not as steep decline in air concentrations with time. In line 4, no apparent change in air concentrations can be observed, but then again, the concentrations were low from the start (Table 2). Line 4 produces mainly diesel fuels low in benzene, partially explaining the low concentrations encountered. In addition, line 4 is the newest production line, most likely benefitting from the use of more recent technical solutions and components. During recent years (2008–2014), the average of benzene air concentrations in all production lines other than line 3 have been consistently less than 10% of the current OEL_8h_ value (Table 4). In line 3, the 95% point is 32% of the OEL_8h_ value. As benzene exposure, for the most part, have been low in all production lines, declining (5%) benzene exposures could be established only in line 5, where the initial exposures were higher than in the more recently built production lines in Porvoo (Table 3). 

TPH air concentrations have declined in all production lines except line 4. However, particularly in production lines 1, 3 and 5, much remains to be done in order to reach TPH air concentrations similar to production line 4: systematically less than 10% of OEL_8h_ (Table 5). Line 4 produces a higher-boiling hydrocarbon mixture (diesel) than production lines 1–4, while line 5 produces solvents and other special products, including fuel oil and bitumen, in addition to diesel. This may in part explain why high concentrations of hydrocarbons with low boiling points may occur there (Table 2 and Table 5). In addition, some of the pumps distributing input, output and side currents, are located in hangars in line 5 as opposed to outdoors in lines 1–4. Similar to benzene, a significant decline in TPH exposure (86%) could be established only in line 5 (Table 3).

### 3.2. Tank Yard, Wastewater Treatment, Maintenance and Technology Center (Porvoo)

These units represent diverse functions with few if any common denominators other than the fact that they all serve production units 1–4. Of these units, only wastewater treatment show a clearly declining trend in air concentrations (Table 2). 

Tank yard is responsible for blending the feeds from the different production lines to finished products and may be susceptible to stray leaks and emissions particularly in association with modifications in finished products, as well as during shut downs and maintenance operations. During the measured time span (1977–2014), benzene as well as TPH air concentrations have occasionally reached values closed to prevailing OEL_8h_ values in the tank yard. Even though only one percent of measurements in the tank yard have exceeded OEL_8h_, this has resulted in average concentrations close to the current OEL_8h_, while the medians are low in comparison (Table 4 and Table 5). During recent years, starting with 2008, the 95% percentile of the air concentrations of both benzene and TPH in the tank yard have remained clearly above 10% of the current OEL_8h_. 

In the wastewater treatment unit, the air concentrations have developed more positively (a decline of 55.99%, Table 2 and Table 3). The wastewater treatment unit is continuously burdened with streams of high hydrocarbon content. Particularly benzene seems to represent a challenge, partially due to polymer production units located in the same factory area as the oil refinery. Benzene concentrations higher than the present OEL_8h_ were recorded relatively frequently up until 2008 but during recent years, benzene air concentrations have usually remained below 10% of the OEL_8h_ (Table 4).

The Technology Center contains R&D units, as well as analytical and measuring services. Different solvents are used or produced in laboratory and/or pilot–scale operations. Benzene exposure has not been an issue for the past ten years in the Technology Center, but the TPH air concentrations have increased from 1977 to 2014. However, since 2004 high concentrations of TPH (more than 50% of current OEL_8h_) have been measured only in conjunction with two isolated incidents not descriptive of average working day exposure: a temporary operational break in an exhaust ventilator in a cool storage room and the uncontrolled purification of equipment using hexane (Table 2, Table 4 and Table 5).

In the maintenance unit, benzene air concentrations—as well as exposure—has been low throughout the years, while TPH air concentrations and exposure show an increase based on the calculations (Table 2 and Table 3). However, TPH air concentrations, and hence exposure, have never been found to exceed 10% of the current TPH OEL_8h_. Highest average working day exposures (10.18 mg/m^3^) have been measured in association with the application of corrosion inhibitors. In other words, TPH exposures in the maintenance functions were fairly low to begin with and have remained so during the measured time interval.

### 3.3. Harbour, and Distribution and Unloading Terminals (Porvoo)

Harbour functions as well as the distribution and unloading of petrochemicals include tasks that yield high air concentrations and are repeated several times during a normal working day. As high immediate benzene concentrations may be relevant to the manifestation of health implications, it is important that all units where benzene emissions is of concern, show a declining trend with time. This has been the case with the benzene air concentrations in the harbour functions (relative decline of measured concentrations 49%), as well as the distribution terminal (relative decline 99%, Table 2). However, in the unloading terminal benzene air concentrations or exposure have not declined, but then again, initial concentrations were lower there (Table 2 and Table 3). Further, since 2008 concentrations exceeding 10% of the current benzene OEL_8h_ has been measured only once in the unloading terminal, from the breathing zone of an operator wearing a half mask.

While hydrocarbon air concentrations show a declining trend with time only in the distribution terminal, the concentrations during recent years have for the most part been low to moderate. The highest TPH concentrations have been recorded in association with the loading of trains, 1.220 mg/m^3^, corresponding to 0.5.110% of the current Finnish OEL_8h_.

In the distribution terminal, air concentrations of both benzene and TPH were high up until 1993. After the maximum concentration of benzene in petrol was lowered from 2 to 1% in 1994, air concentrations have been lower. In the loading of ships, recovery of gaseous emissions was taken into use in 2014, the effect of which will be seen during upcoming years.

### 3.4. Naantali: Tank Yard, Harbour, Distribution Terminal, Wastewater Treatment and Maintenance

In the Naantali production line as well as in the tank yard and wastewater treatment functions, we see a clear decline in the air concentrations with time, resulting in a reduction of exposure as well (Table 2 and Table 3). Most other measured concentrations in these units have reduced throughout the years as well. Exceptions to this trend are benzene air concentrations in the distribution terminal, benzene exposure and air concentrations in the maintenance functions, benzene exposure in the harbour, and TPH exposure and air concentrations in the harbour. In previous years, benzene and particularly TPH OEL_8h_ values have frequently been exceeded in the tank yard as well as in the distribution terminal. During recent years, 2005 and onwards, concentrations higher than OEL_8h_ have been recorded only in the distribution terminal. In units other than the distribution terminal, including the wastewater treatment unit, measured concentrations of benzene as well as other hydrocarbons have for the most part been fairly low (Table 4 and Table 5).

In the distribution terminal, high concentrations of TPH, have been recorded in conjunction with vehicle dispatching. Measurements describing average working day exposure have, however, yielded low results in all units.

## 4. Discussion

In general, benzene and hydrocarbon exposure in Finnish industries have declined during the past decades. For instance, according to the FINJEM-database, the number of workers exposed to benzene have decreased more than 10-fold concurrently with the present study: from 10,013 (1960–1984) to 593 (2007–2009) [23]. On average, benzene exposure in industrial workplaces in Finland declined 52% and total volatile hydrocarbon exposure (category two solvent petrols) 99% during 1994–2011, as estimated from FIOH service measurements. Published reports by the CONCAWE indicate a similar development in oil refining in general [24]. In investigations on the benzene exposure at ExxonMobil refineries in Louisiana and Texas, up until 2006, similar mean air concentrations were reported, i.e., 0.01–0.4 ppm [4,9,10]. In those studies, from 1976 to 2007 (Texas) and 1977–2005 (Louisiana), declining exposures were established for job categories “laboratory technician” (Texas) and “process technician”, excluding waste treatment area (Louisiana). However, no precise quantitative estimates are available enabling a comparison to the exposure measurements performed by Neste Ltd. during the whole time frame 1977–2014.

In the present study, benzene air concentrations declined in 11 out of 17 units, six of which were associated with a significant decline in benzene exposure as well. Benzene air concentrations declined across all units on average by 46%. This amounts to an average decline of 1.7% per year. TPH air concentrations declined in 12 out of 17 units, seven of which were associated with a decline in exposure. Across all units, the average decline in TPH air concentrations was 49%, corresponding to 1.3% per year.

In all sites apart from the Porvoo harbor and ships, benzene median values or the level at the end points of the regression curves are at or below 0.1 ppm. Using an old SCOEL (1991) estimate on cancer risk this represents a cancer risk in the range of 0.05–0.66 per 1000 in 40 years occupational exposure [6]. This risk estimate is based on the Crump and Allen analysis of so-called Plioform cohort and assumes linear dose response [25]. It should, however, be noticed that there are large uncertainties related to the magnitude of benzene caused cancer risk at low exposure levels. Different analyses on the dose-response of the carcinogenicity of benzene at these low levels has been published since then. For example, the German AGS derived so-called “tolerable cancer risk level” of 0.6 ppm resulting in four extra cancers per 1000 exposed workers, and an “acceptable cancer risk level” of 0.06 ppm resulting in four extra cancers per 10,000 exposed workers based on linear extrapolation from the dose-response data derived from several epidemiological studies. On the other hand, Dutch DECOS Committee recently proposed an OEL of 0.2 ppm for benzene based on the haematotoxicity of benzene [7]. Benzene was considered as a non-stochastic genotoxic carcinogen for which a threshold for carcinogenicity could be established. Since haematotoxicity often precedes benzene caused leukemia, preventing haematotoxicity was considered to minimise the risk of leukaemia [7]. In US, ACGIH has concluded that at a TLV level of 0.5 ppm odds of death from leukemia due to occupational benzene exposure would be indistinguishable from the odds of death from leukemia of non-exposed workers [26]. This conclusion was based on Plioform data. Overall, although no definitive conclusions can be made on the cancer risks at the levels below 0.1 ppm, it is advisable to minimize the exposure to benzene as low as reasonably achievable. In the wastewater treatment units a 7.28-fold decrease in exposure levels during the follow-up period was recorded. This is likely to have a significant impact on cancer risk as well. In most sites (10 out of 17) however, benzene exposure was below 0.1 ppm to begin with (<10% of OEL_8h_). In these units, no statistically significant decline in exposure could be recorded, regardless of whether the air concentrations declined or not (Table 2 and Table 3).

As the present investigation spans nearly four decades, numerous outside factors have affected work related exposure to hydrocarbons in the refineries. Such factors include (effect, either positive or negative, in captions): lowering the maximum benzene concentration allowed in petrol gradually: 3.5% in 1980, 2.5% in 1985, 2% 1991, 1% in 1994 (−); increase of benzene-rich effluents from polymer production units to wastewater treatment plant in Porvoo (+); changes in production capacity and product portfolio (±); technological progress and better process engineering techniques (−); new legislation concerning safety and risk management as well as cancer registry (−); stricter OEL_8h_ values (−). In addition, measuring techniques and analysis methods of organic emissions have improved, and new techniques and equipment have become available enabling online measurement of air concentrations and in some cases even exposure. Consequently, it is likely that in 2014 we get a more reliable picture of air concentrations and work related exposure at the refineries, than we did in 1977.

In managing exposure, particular effort should be placed on control of emissions at the source [27]. In the refineries, such measures include careful planning of new production lines and process technological solutions. However, much can and has been done to improve conditions in older units as well, as can be seen from the development of air concentrations in production line 5 (Naantali). A decline in air concentrations in production line 5 as well as other units critical with respect to exposure to volatile hydrocarbons have been made possible through the consistent implementation of exposure control measures: (1) Application of systematic on-line measurements of VOC leaks using personal LDAR monitors starting 1997; (2) Hydration of benzene starting 1993; (3) Distillation unit to remove benzene for the purpose of polymer production starting 1993; (4) Floating roofs in petrol containers starting 1980s and, starting 1990, in all containers containing volatiles; (5) Superior valves and seals starting 1990s; (6) Hermetic pumps starting 1990s; (7) Recovery of loading gases in the distribution terminals (1990); (8) Recovery of loading gases in the dock starting January 2014; (9) Better recovery of benzene starting 2000; (10) Installment of a torch in doc 8 and unloading terminals (Porvoo), 2010; (11) Gradual improvement of the efficiency of the torches; (12) Termination and/or cutback of the combustion of coke formed in catalysis and distillation. Moreover, from 1977–2014, many important measures have been incorporated into the safety management system of the refineries, to help to prevent and manage exposure to chemicals: (1) Systematic evaluation of work-related risks and exposure; (2) Auditing practices; (3) Recording and investigation of disturbances, with the aim to improve managing practices; (4) Development of online monitoring systems for volatile hydrocarbons, including benzene; (5) Training programs and work-permit practices; (6) Improvements in practices pertaining to protective equipment; (7) Occupational safety card.

Providing other control measures are inadequate to prevent excessive exposure, the use of protective equipment is necessary. Neste Ltd. has a corporate policy for the use of protective equipment. Enforcing and supporting the use of protective equipment is often problematic. In fact, it has been suggested that “personal protective equipment should be used only sparingly and under appropriate circumstances, and never as a substitute for more reliable and effective engineering or administrative controls” [28]. In other words—and with reference to the Neste Ltd. policy for the use of protective equipment—the goals should be to lower benzene air concentrations below 10% of the current benzene OEL_8h_ and TPH air concentrations below 50% of the current TPH OEL_8h_. This would help to focus the need for protective equipment mainly to infrequent tasks short in duration.

## 5. Conclusions

In conclusion, air concentrations of TPH and benzene have generally declined with time (1977–2014). In more than half of the units where a decrease in air concentrations were established, this decrease was accompanied by a decrease in exposure. When a decrease in exposure could not be established, the exposure was without exception less than 10% of the current OEL_8h_ to begin with. 

However, there is still a need for improvements in many of the units as benzene concentrations below 0.1 ppm may still possess a risk to exposed workers. It should also be noted that according to some studies (repeated) short term (≤15 min) peak exposures may play a role in benzene carcinogenesis [29]. Therefore, consideration should be given also to minimizing leaks resulting in short term peak exposures. Further, benzene and/or TPH air concentrations did not improve in 8 out of 17 of the units, some of which are associated with average air concentrations exceeding 10% of the relevant OEL_8h_ value (Table 4 and Table 5). Considering the harmfulness of the exposure agents in question, the average 8 h benzene exposure and air concentrations of benzene should as a minimum not exceed 10% of the OEL_8h_, while the average air concentrations to TPH as well as TPH exposure should be kept below 50% of the OEL_8h_. In addition, in no circumstances should the workers be continually exposed to concentrations exceeding OEL_8h_ when not protected. With this in mind, and considering that the OEL_8h_ of benzene will possibly be lowered in the near future, we suggest that when possible, the highest benzene air concentrations should be lowered in the following operations and units (in order of priority, see Table 4): (1) Harbour and ships (Porvoo); (2) Unloading terminal (Porvoo); (3) the tank yards (Porvoo and Naantali); (4) Wastewater treatment (Naantali); (5) Production line 3 (Porvoo). Also, based on recent (2008–2014) measurements of TPH air concentrations and exposure (Table 5), we suggest that further exposure control measures of hydrocarbon exposure should be directed to the following units (in order of priority): (1) Unloading terminal (Porvoo); (2) Harbour (Naantali); (3) Distribution terminal (Naantali).

Regardless of the unit or exposure agent, the maintenance shut downs of the refineries (in Porvoo 1993, 1997, 2001, 2005, 2010; in Naantali 2000, 2006, 2012) have generally been associated with clear peaks in the air concentrations. Consequently, particular effort should be placed on safe working methods pertaining to shutdowns as well as the use and condition of respiratory protective equipment of subcontractors and in-house workers alike. Also, while recovery of loading gases in the distribution terminals and docks will most likely lower air concentrations in these units, the connection and detachment of hoses continue to be challenging from the point of view of controlling excessive exposure.

Benzene and hydrocarbon air concentrations in some of the units investigated will most likely continue to have room for improvement for years to come. In the Neste refineries, personal LDAR-monitors and on-line measurements, monitoring campaigns and risk assessment procedures, availability of protective equipment as well as training programs are effective tools in the monitoring and management of exposure. However, ensuring safe working conditions and working methods will continue to require persistent work. Many problematic work tasks and situations will still have to be addressed in order to improve high air concentrations and further lessen the need for protective equipment. Also, as an instrument in lowering exposure with recent risk estimates in mind, methods to estimate exposure at the refineries need to be improved to enable measuring benzene concentrations <0.1 ppm.

## Figures and Tables

**Table 1:** Finnish Occupational Exposure Limit (OEL_8h)_ for benzene and TPH compounds, 1960–2014.

Year	OEL_8h_	
	TPH Compounds (mg/m^3^)	Benzene (ppm)
2014	200	1
2012	200	1
2009	200	1
2007	200 ^a^	1
2005	770	1
2002	770	1
2000	770	1
1998	770	5
1996	770	5
1993	770	5
1987	770 ^b^	5 ^f^
1981	780 ^c^	5
1977	1150	10
1972	1150 ^d^	10
1960	2000 ^e^	25

^a^ Aromatics content 1–25%, hexane <1%; ^b^ aromatics <20%, boiling point ≥110 °C; ^c^ aromatics 20%; ^d^ aromatics <20%; ^e^ naphta; ^f^ binding limit value staring 1987.

**Table 2:** Air concentrations, measurements and statistics.

	Unit (Number of Presently Exposed Workers in Brackets)	Exposure Agent	Timespan	Number of Measurements	Measurements > OEL (%)	P (Critical ^3^ Value of ρ_x,y_)	R^2^ (Coefficient of Determination)	Pearson Correlation (ρ_x,y_)	ρ_x,y_ ≥ P	Reduction (%)	Starting Point ^4^ (mg/m^3^ or ppm)	End Point ^5^ (mg/m^3^ or ppm)
1	Porvoo harbour and ships	Benzene	1977–2014	659	1	0.076	0.0175	0.132	true	49	2.75	1.41
	(50–100)	Hydrocarbons	1977–2014	659	0	0.076	2.00 × 10^−7^	0.000	false	±0	4.00 ^1^	4.00 ^1^
2	Porvoo distribution terminal	Benzene	1977–2011	368	7	0.102	0.1082	0.329	true	>99	4.37	<0.10 ^2^
	(10–20)	Hydrocarbons	1977–2011	368	1	0.102	0.1569	0.396	true	>99	245	<0.10 ^2^
3	Porvoo unloading terminal	Benzene	1998–2014	77	1	0.224	0.0039	0.062	false	±0	<0.10 ^1,2^	<0.10 ^1,2^
	(20–50)	Hydrocarbons	1998–2014	77	0	0.224	0.0039	0.062	false	±0	5.00 ^1^	5.00 ^1^
4	Porvoo. production line 1	Benzene	1977–2014	366	0	0.103	0.0485	0.220	true	54	0.24	0.11
	(100–150)	Hydrocarbons	1977–2014	366	0	0.103	0.0533	0.231	true	65	3.90	1.36
5	Porvoo. production line 2	Benzene	1977–2014	231	0	0.129	0.0264	0.162	true	15	0.08	0.07
	(100–150)	Hydrocarbons	1977–2014	231	0	0.129	0.0259	0.161	true	>99	1.87	<0.10
6	Porvoo. production line 3	Benzene	1977–2011	718	0	0.073	0.0428	0.207	true	71	0.13	<0.10 ^2^
	(100–150)	Hydrocarbons	1977–2011	718	0	0.073	0.0120	0.110	true	42	5.57	3.23
7	Porvoo. production line 4	Benzene	2009–2013	42	0	0.304	2.00 × 10^−13^	0.000	false	±0	<0.10 ^1,2^	<0.10^1 ,2^
	(100–150)	Hydrocarbons	2009–2013	42	0	0.304	0.0312	0.177	false	±0	0.50 ^1^	0.50 ^1^
8	Porvoo. tank yard	Benzene	1977–2014	175	1	0.148	0.0027	0.052	false	±0	0.10 ^1^	0.10 ^1^
	(50–100)	Hydrocarbons	1977–2014	175	1	0.148	4.00 × 10^−5^	0.006	false	±0	2.00 ^1^	2.00 ^1^
9	Porvoo. wastewater treatment	Benzene	1979–2014	726	4	0.073	0.0523	0.229	true	>99	4.48	<0.10
	(20–50)	Hydrocarbons	1979–2014	726	0	0.073	0.0068	0.082	true	55	19.85	8.90
10	Porvoo. maintenance	Benzene	1977–2008	38	0	0.320	0.0248	0.157	false	±0	<0.10 ^1,2^	<0.10 ^1,2^
	(100–150)	Hydrocarbons	1977–2008	38	0	0.320	0.3647	0.604	true	>−99	<0.10 ^2^	6.67
11	Porvoo. Technology center	Benzene	1975–2014	222	0	0.132	0.0490	0.221	true	65	0.43	0.14
	(20–50)	Hydrocarbons	1975–2014	222	1	0.132	0.0174	0.132	true	>−99	1.22	69.19
12	Naantali. production line 5	Benzene	1981–2011	504	1	0.087	0.0449	0.212	true	58	0.21	<0.10 ^2^
	(50–100)	Hydrocarbons	1981–2011	635	0	0.078	0.1101	0.332	true	98	69.25	1.25
13	Naantali. tank yard	Benzene	1981–2011	158	1	0.156	0.0827	0.288	true	76	0.57	0.14
	(20–50)	Hydrocarbons	1981–2011	164	1	0.153	0.0555	0.236	true	92	155	11.71
14	Naantali. distribution terminal	Benzene	1981–2013	86	1	0.212	0.0168	0.130	false	±0	19.00 ^1^	19.00 ^1^
	<10	Hydrocarbons	1981–2013	86	0	0.212	0.0616	0.248	true	85	85.15	12.88
15	Naantali. harbour	Benzene	1981–2011	26	0	0.388	0.2249	0.474	true	>99	0.27	<0.10 ^2^
	(10–20)	Hydrocarbons	1981–2011	26	0	0.388	0.1183	0.344	false	±0	36.00 ^1^	36.00 ^1^
16	Naantali. wastewater treatment	Benzene	1981–2014	121	2	0.179	0.2411	0.491	true	>99	1.40	<0.10 ^2^
	(10–20)	Hydrocarbons	1981–2014	121	2	0.179	0.2648	0.515	true	>99	345	<0.10 ^2^
17	Naantali. maintenance	Benzene	1980–2011	59	0	0.256	0.0497	0.223	false	±0	<0.10 ^1,2^	<0.10 ^1,2^
	(20–50)	Hydrocarbons	1980–2011	65	0	0.244	0.0638	0.253	true	>99	247.92	<0.10 ^2^

^1^ Medians; ^2^ below limit of quantitation; ^3^ critical value of Pearson’s correlation coefficient; ^4^ starting point of concentration curve corresponding to the initial date of sampling, the unit is either mg/m^3^ (hydrocarbons) or ppm (benzene); ^5^ end point of concentration curve corresponding to the last date of sampling, the unit is either mg/m^3^ (hydrocarbons) or ppm (benzene).

**Table 3:** Inhalation exposure, measurements and statistic.

	Unit (Number of Presently Exposed Workers in Brackets)	Exposure Agent	Timespan	Number of Measurements	Measurements > OEL (%)	P (Critical ^3^ Value of ρ_x,y_)	R^2^ (Coefficient of Determination)	Pearson Correlation (ρ_x,y_)	ρ_x,y_ ≥ P	Reduction (%)	Starting Point ^4^ (mg/m^3^ or ppm)	End Point ^5^ (mg/m^3^ or ppm)
1	Porvoo harbour and ships	Benzene	1977–2014	189	3	0.143	0.048	0.2191	true	43	2.96	1.69
	(50–100)	Hydrocarbons	1977–2014	189	0	0.143	3.00 × 10^−6^	0.0017	false	-	4.00 ^1^	4.00 ^1^
2	Porvoo distribution terminal	Benzene	1977–2011	121	2	0.179	0.1122	0.3350	true	51	0.20	<0.10 ^2^
	(10–20)	Hydrocarbons	1977–2011	121	0	0.179	0.169	0.4111	true	>99	116.56	<0.10 ^2^
3	Porvoo unloading terminal	Benzene	1998–2014	38	0	0.320	0.0032	0.0566	false	-	<0.10 ^1,2^	<0.10 ^1,2^
	(20–50)	Hydrocarbons	1998–2014	38	0	0.320	3.00 × 10^−5^	0.0055	false	-	5.00 ^1^	5.00 ^1^
4	Porvoo. production line 1	Benzene	1977–2014	90	0	0.207	0.0373	0.1931	false	-	<0.10 ^1,2^	<0.10 ^1,2^
	(100–150)	Hydrocarbons	1977–2014	90	0	0.207	0.0047	0.0686	false	-	1.00 ^1^	1.00 ^1^
5	Porvoo. production line 2	Benzene	1977–2014	86	0	0.212	1.00 × 10^−13^	0.0000	false	-	<0.10 ^1,2^	<0.10 ^1,2^
	(100–150)	Hydrocarbons	1977–2014	86	0	0.212	0.0024	0.0490	false	-	1.00 ^1^	1.00 ^1^
6	Porvoo. production line 3	Benzene	1977–2011	165	0	0.153	0.006	0.0775	false	-	<0.10 ^1,2^	<0.10 ^1,2^
	(100–150)	Hydrocarbons	1977–2011	165	0	0.153	0.0049	0.0700	false	-	1.50 ^1^	1.50 ^1^
7	Porvoo. production line 4	Benzene	2009–2013	22	0	0.423	3.00 × 10^−13^	0.0000	false	-	<0.10 ^1,2^	<0.10 ^1,2^
	(100–150)	Hydrocarbons	2009–2013	22	0	0.423	0.0435	0.2086	false	-	0.50 ^1^	0.50 ^1^
8	Porvoo. tank yard	Benzene	1977–2014	142	1	0.165	0.002	0.0447	false	-	0.10 ^1^	0.10 ^1^
	(50–100)	Hydrocarbons	1977–2014	142	1	0.165	0.0021	0.0458	false	-	2.30 ^1^	2.30 ^1^
9	Porvoo. wastewater treatment	Benzene	1979–2014	211	0	0.135	0.0563	0.2373	true	85	0.67	<0.10 ^2^
	(20–50)	Hydrocarbons	1979–2014	211	0	0.135	0.0004	0.0200	false	-	2.00 ^1^	2.00 ^1^
10	Porvoo. maintenance	Benzene	1977–2008	38	0	0.320	0.0248	0.1575	false	-	<0.10 ^1,2^	<0.10 ^1,2^
	(100–150)	Hydrocarbons	1977–2008	38	0	0.320	0.3647	0.6039	true	>−99	<0.10 ^2^	6.67
11	Porvoo. Technology center	Benzene	1975–2014	114	0	0.184	0.0087	0.0933	false	-	<0.10 ^1,2^	<0.10 ^1,2^
	(20–50)	Hydrocarbons	1975–2014	114	1	0.184	0.0158	0.1257	false	-	4.50 ^1^	4.50 ^1^
12	Naantali. production line 5	Benzene	1981–2011	129	0	0.173	0.0351	0.1873	true	5	0.11	<0.10 ^2^
	(50–100)	Hydrocarbons	1981–2011	143	0	0.164	0.027	0.1643	true	86	31.65	4.48
13	Naantali. tank yard	Benzene	1981–2011	56	2	0.263	0.0703	0.2651	true	82	0.55 ^1^	<0.10 ^1,2^
	(20–50)	Hydrocarbons	1981–2011	56	0	0.263	0.1738	0.4169	true	>99	75.45	<0.10 ^2^
14	Naantali. distribution terminal	Benzene	1981–2013	27	0	0.381	0.2002	0.4474	true	76	0.41	<0.10 ^2^
	<10	Hydrocarbons	1981–2013	27	0	0.381	0.3087	0.5556	true	>99	178.06	<0.10 ^2^
15	Naantali. harbour	Benzene	1981–2011	7	0	0.754	0.2543	0.5043	false	-	0.10 ^1^	0.10 ^1^
	(10–20)	Hydrocarbons	1981–2011	7	0	0.754	0.0818	0.2860	false	-	64.00 ^1^	64.00 ^1^
16	Naantali. wastewater treatment	Benzene	1981–2014	21	5	0.433	0.5588	0.7475	true	96	2.84	<0.10 ^2^
	(10–20)	Hydrocarbons	1981–2014	21	10	0.433	0.6374	0.7984	true	>99	842.76	<0.10 ^2^
17	Naantali. maintenance	Benzene	1980–2011	58	0	0.259	0.0544	0.2332	false	-	<0.10 ^1,2^	<0.10 ^1,2^
	(20–50)	Hydrocarbons	1980–2011	64	0	0.246	0.0662	0.2573	true	>99	54.72	<0.10 ^2^

^1^ Medians; ^2^ below limit of quantitation; ^3^ critical value of Pearson’s correlation coefficient; ^4^ starting point of concentration curve corresponding to the initial date of sampling, the unit is either mg/m^3^ (hydrocarbons) or ppm (benzene); ^5^ end point of concentration curve corresponding to the last date of sampling, the unit is either mg/m^3^ (hydrocarbons) or ppm (benzene).

**Table 4:** Development and end points of benzene air concentrations and end points of benzene exposure.

Unit	Number of Measurements	Median, Air Concentrations (ppm)	Average, Air Concentrations (ppm)	95% Percentile, Air Concentration ^2^ (ppm)	95% Percentile, Exposure ^2^ (ppm)
Porvoo, production line 1	160	<0.10 ^2^	<0.10 ^2^	0.10/<0.10	<0.10
Porvoo, production line 2	60	<0.10 ^2^	<0.10 ^2^	0.10/<0.10	<0.10
Porvoo, production line 3	193	<0.10 ^2^	0.19 ^2^	0.30/<0.10	<0.10
Porvoo, production line 4	42	<0.10 ^2^	<0.10 ^2^	<0.10/<0.10	<0.10
Naantali, production line 5	134	<0.10 ^2^	<0.10 ^2^	0.10/<0.10	<0.10
Porvoo, tank yard	328	<0.10 ^1^	1.0 ^1^	0.20/-	-
Porvoo, wastewater treat.	981	0.10 ^1^	5.0 ^1^	0.10/<0.10	<0.10
Porvoo, maintenance	75	<0.10 ^1^	0.73 ^1^	<0.10/0.10	<0.10
Porvoo, Technology center	306	0.10 ^1^	0.20 ^1^	<0.10/<0.10	0.10
Porvoo harbour and ships	840	<0.10 ^1^	0.83 ^1^	0.40/0.45	0.45
Porvoo distr. terminal	718	0.30 ^1^	2.0 ^1^	<0.10/<0.10	<0.10
Porvoo unloading terminal	188	<0.10 ^1^	1.1 ^1^	0.20/<0.10	<0.10
Naantali, tank yard	212	0.10 ^1^	0.52 ^1^	0.18/0.27	0.27
Naantali, distr. terminal	243	<0.10 ^1^	0.27 ^1^	0.10/<0.10	<0.10
Naantali, harbour	46	<0.10 ^1^	0.20 ^1^	-/-	-
Naantali, wastew. treat.	167	<0.10 ^1^	0.35 ^1^	0.14/-	-
Naantali, maintenance	81	<0.10 ^1^	0.14 ^1^	-/-	-

^1^ average (median) spanning the entire time frame; ^2^ 2008–2014.

**Table 5:** Development and end points of hydrocarbon air concentrations and end points of hydrocarbon exposure.

Unit	Number of Measurements	Median, Air Concentrations (ppm)	Average, Air Concentrations (ppm)	95% Percentile, Air Concentration ^2^ (mg/m^3^)	95% Percentile, Exposure ^2^ (mg/m^3^)
Porvoo, production line 1	168	1 ^2^	9 ^2^	39/15	15
Porvoo, production line 2	65	1 ^2^	3 ^2^	10/6	6
Porvoo, production line 3	198	1 ^2^	4 ^2^	79/3	3
Porvoo, production line 4	42	0.5 ^2^	2 ^2^	6/5	5
Naantali, production line 5	134	4 ^2^	28 ^2^	92/62	62
Porvoo, tank yard	328	2 ^1^	32 ^1^	76/-	-
Porvoo, wastewater treat.	981	2 ^1^	33 ^1^	4/4	4
Porvoo, maintenance	75	1 ^1^	19 ^1^	17/17	17
Porvoo, Technology center	306	4 ^1^	35 ^1^	386 ^3^/33	33
Porvoo harbour and ships	840	4 ^1^	18 ^1^	56/18	18
Porvoo distr. terminal	718	24 ^1^	129 ^1^	17/2	2
Porvoo unloading terminal	188	5 ^1^	40 ^1^	96/44	44
Naantali, tank yard	212	30 ^1^	157 ^1^	56/51	51
Naantali, distr. terminal	243	24 ^1^	174 ^1^	477/2	2
Naantali, harbour	46	35 ^1^	147 ^1^	-/-	-
Naantali, wastew. treat.	167	10 ^1^	89 ^1^	75/-	-
Naantali, maintenance	81	37 ^1^	10 ^1^	-/-	-

^1^ average (median) spanning the entire time frame; ^2^ 2008–2014; ^3^ high exposures have been associated with two working tasks short in duration; problems have currently been resolved.
